# Identification of Usutu Virus Africa 3 Lineage in a Survey of Mosquitoes and Birds from Urban Areas of Western Spain

**DOI:** 10.1155/2023/6893677

**Published:** 2023-02-22

**Authors:** Daniel Bravo-Barriga, Martina Ferraguti, Sergio Magallanes, Pilar Aguilera-Sepúlveda, Francisco Llorente, Elisa Pérez-Ramírez, Ana Vázquez, Fátima Guerrero-Carvajal, María Paz Sánchez-Seco, Miguel Ángel Jiménez-Clavero, Carlos Mora-Rubio, Alfonso Marzal, Eva Frontera, Florentino de Lope

**Affiliations:** ^1^Universidad de Extremadura, Facultad de Veterinaria, Departamento de Sanidad Animal, Parasitología, Avda. Universidad s/n, Cáceres 10003, Spain; ^2^Universidad de Extremadura, Facultad de Biología, Departamento de Anatomía, Biología celular y Zoología, Avenida de Elvas s/n, Badajoz 06006, Spain; ^3^University of Amsterdam, Department of Theoretical and Computational Ecology (TCE), Institute for Biodiversity and Ecosystem Dynamics (IBED), Science Park 904, Amsterdam 1098 XH, Netherlands; ^4^Estación Biológica de Doñana (EBD-CSIC), Departamento de Ecología de los Humedales, Avda. Américo Vespucio 26, Sevilla 41092, Spain; ^5^Centro de Investigación en Sanidad Animal (CISA-INIA), CSIC, Valdeolmos 28130, Spain; ^6^Laboratorio de Arbovirus y Enfermedades Víricas Importadas, CNM-Instituto de Salud Carlos III, Majadahonda 28220, Madrid, Spain; ^7^Centro de Investigación Biomédica en Red de Epidemiología y Salud Pública (CIBERESP), Madrid, Spain; ^8^Centro de Investigación Biomédica en Red de Enfermedades Infecciosas (CIBERINFEC), Madrid, Spain; ^9^Grupo de Investigación y Sostenibilidad Ambiental, Universidad Nacional Federico Villarreal, Lima, Peru

## Abstract

Usutu virus (USUV) is an emerging zoonotic arbovirus that has caused an increasing number of animal and human cases in Europe in recent years. Understanding the vector species and avian hosts involved in the USUV enzootic cycle in an area of active circulation is vital to anticipate potential outbreaks. Mosquitoes were captured in 2020, while wild birds were sampled in both 2020 and 2021 in Extremadura, southwestern Spain. The presence of USUV in the mosquito vectors was assessed by a real-time reverse transcription-polymerase chain reaction (rRT-PCR) assay and confirmed by sequencing amplicons from two generic RT-PCR sets for flaviviruses. Sequences were analysed phylogenetically. Bird sera were screened for flavivirus antibodies with a blocking ELISA kit and subsequently tested for virus-specific antibodies with a micro-virus-neutralization test. Overall, 6,004 mosquitoes belonging to 13 species were captured, including some well-known flavivirus vectors (*Culex pipiens*, *Cx. perexiguus,* and *Cx. univittatus*). Of the 438 pools tested, USUV was detected in two pools of *Cx. pipiens.* Phylogenetic analysis using a fragment of the NS5 gene assigned the USUV detected the Africa 3 lineage. Out of 1,413 wild birds tested, USUV-specific antibodies were detected in 17 birds (1.2%, 10 males and 7 females) from eight species. The first detection of USUV Africa 3 lineage in mosquitoes from Spain, together with serologically positive resident wild birds in urban and rural areas, indicates active circulation and a possible risk of exposure for the human population, with necessity to establish specific surveillance plans.

## 1. Introduction

Usutu virus (USUV) is a mosquito-borne flavivirus (family Flaviviridae), closely related antigenically and genetically to West Nile virus (WNV) and belonging to the Japanese encephalitis serocomplex. In nature, USUV is maintained in a bird-mosquito-bird enzootic cycle [1]. However, the virus has also been detected sporadically in humans, horses, and other mammals, acting as incidental hosts (dead-end hosts, i.e., they can be infected but do not transmit the infection), and in a limited number of cases, they can suffer neuroinvasive disease and USUV fever [2, 3].

The first detection of USUV was in *Culex neavei* mosquitoes from South Africa in 1959, in a close area to the Usutu River in Swaziland [4]. Afterwards, the virus was retrospectively detected in archived tissue samples from birds from Italy collected in 1996 [5]. In 2001, USUV was identified as the causative agent of massive bird mortality in Austria [6]. Since then, USUV has been detected in several European countries in mosquitoes, birds, various mammals (horses, bats, ruminants, dogs, and wild boars), and humans [1, 3, 7–9].

In recent years, the understanding of the ecology, epidemiology, and distribution of USUV has improved due to an increase in its detection as a result of enhanced flavivirus surveillance and research activities in Europe [3, 10]. So far, eight lineages of USUV have been described, five European lineages (Europe 1–5) and three African lineages (Africa 1–3) [11]. The great diversity of lineages circulating in Europe suggests different introductions from Africa, as well as a continuous geographical spreading across the continent and colonization of new ecological niches [10, 12]. The rapid European expansion of the virus, together with several reports of infection cases or neuroinvasive diseases in humans, has confirmed USUV as an emerging zoonotic virus of public health significance [1, 3].

In Spain, USUV-specific antibodies have been detected from different species of wild birds, horses, and red deer [13]. In mosquitoes, USUV has been identified in *Culex pipiens* in 2006 (northeastern Spain) and in *Culex perexiguus* in 2009 (southern Spain), both belonging to the USUV Africa 2 lineage [14, 15]. Another USUV sequence was also obtained in 2012 from a song thrush (*Turdus philomelos*) related to a Senegalese and Central European sequences, although without a clear classification [16, 17]. In the region of this study, Extremadura (southwestern Spain), USUV antibodies were detected for the first time in 2017–2019 in horses and wild birds [18–20], but evidence from mosquitoes or humans has not been reported yet.

To date, field studies focusing on USUV enzootic cycle are scarce. USUV shares important features with WNV, including a degree of overlapping ranges of vector species and avian hosts, as well as some cross-reactivity in diagnostic methods. Hence, combined surveillance and control programs of both viruses offer important benefits, contributing to improve the understanding of their epidemiology and the potential interactions which may affect the transmission of both pathogens under natural conditions. Here, we report the concomitant detection of the USUV genome in mosquitoes and USUV antibodies in avian hosts, confirming the presence of an enzootic cycle in an area close to urban centers from southwestern Spain.

## 2. Materials and Methods

### 2.1. Study Area

**Figure 1:**
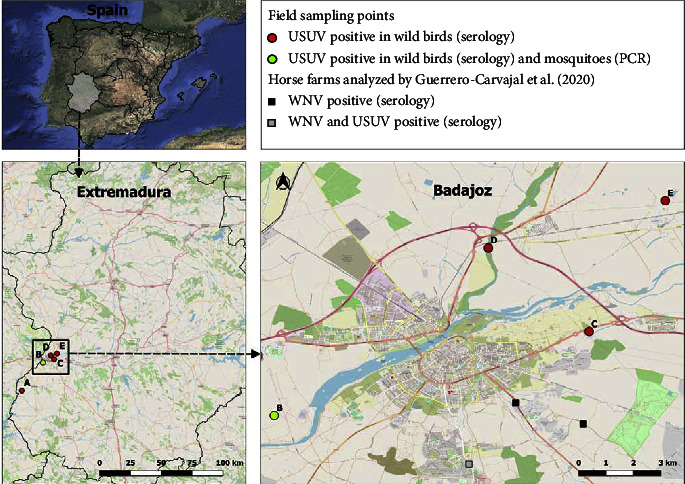
Geographical distribution and results of USUV by serology and PCR in the five sampling points (A: Asesera, B: Rincón de Caya, C: Bótoa, D: Gévora, and E: Sagrajas) in Extremadura, Spain. This map was created by the QGIS geographic information system, version 3.22.0 (2021) (QGIS association, https://www.qgis.org/es/site).

Five sampling sites were selected in Extremadura (southwestern Spain) with suitable habitats for wild birds and mosquitoes. One in a rural location (A: Asesera, 38°39′15″N, 7°13′1″W) and four close to the city of Badajoz (B: Rincón de Caya (38°51′31″N, 7°1′44″W), C: Bótoa (38°53′10″N, 6°55′32″W), D: Gévora (38°54′49″N, 6°57′31″W), and E: Sagrajas (38°55′45″ N, 6°54′2″W)) (Figure 1). Badajoz is the largest urban nucleus (152,764 inhabitants) of the Autonomous Community of Extremadura. Most of the sampling sites were located close to the border with Portugal and the Guadiana River. Extremadura region has a Mediterranean climate classified as Csa (hot dry summer) according to the Köppen climate classification.

### 2.2. Sampling of Mosquitoes and Wild Birds

Mosquitoes were collected from May to November 2020. Entomological sampling was carried out every 40 days, and each trap was set from sunset to early morning, during a minimum of 12 hours. The combination of different traps (i.e., BG-Sentinel traps, CDC miniature light-traps, both containing CO_2_ as bait, and gravid traps baited with hay infusion) ensured a more complete sampling of the vector community present in the area, with a greater diversity of species and catch density [21]. On the following morning, mosquitoes were frozen in the field with dry ice and transported to the laboratory for their morphological identification and molecular analysis.

Morphological identifications at species level were based on broadly used identification keys [22]. The whole process was carried out under binocular magnifying glass with frozen plates to ensure the maintenance of the cold chain. Mosquitoes were grouped by species, collection site, and dates in pools of 1–25 individuals and conserved in the MEM culture medium. Males were equally identified and classified to be considered in abundance estimates but not analysed for USUV detection.

Molecular analyses were performed to confirm the identification of epidemiologically important mosquito species that are difficult to identify morphologically. For this purpose, genomic DNA (gDNA) was extracted from the legs of individuals using GeneJET™ Genomic DNA Purification Kit (Thermo Scientific Inc., reference #K0722) following the manufacturer's instructions. The cytochrome oxidase 1 (*cox*1) gene was partially amplified using the primer set LCO1490 and HCO2198 following the PCR protocol described previously [23]. The amplified product was sequenced on an ABI 3130 genetic analyser (provided by the Service of Bioscience Applied Techniques of the University of Extremadura, SAIUEx), and the sequences were edited through BioEdit Sequence Alignment Editor (version 7.2.5, Carlsbad, CA, U.S.A.). Identification of mosquitoes to species level was obtained by taking into account the highest similarity, using BLASTn (https://blast.ncbi.nlm.nih.gov/Blast.cgi) and the Barcode of Life Data Systems-v4 databases. The respective nucleotide sequences obtained were deposited in the DNA Data Bank of Japan (DDBJ: https://www.ddbj.nig.ac.jp/index-e.html) under accession no. LC659916–8.

Wild birds were captured using mist-nets from February to December 2020 and from March to May 2021. Each bird was ringed with a numbered metal ring, and its age and sex were determined when possible, according to their plumage characteristics and skull ossification [24]. For each individual, a blood sample was extracted from the jugular vein using sterile insulin syringes. The volume of extracted blood varied depending on the body size of each bird and never exceeded 1% of their body mass. Blood samples were transferred to sterile Eppendorf tubes and preserved in cold boxes during the field work. In the laboratory, the samples were kept at 4°C and centrifuged within 24 h after sampling for 10 min at 11,000 rpm (11.2 g) to separate serum and cellular fractions, which were frozen at −80 or −20°C, respectively. Birds were immediately released unharmed at the site of capture after manipulation.

### 2.3. Detection of the Flavivirus Genome in Mosquitoes and Phylogenetic Analyses

Viral RNA was extracted from mosquito pools using a MagMax™ Pathogen RNA/DNA kit (ThermoFisher®), according to the manufacturer's protocol (Pub. no. 4463379). At least one negative control and a triplex positive extraction control (for WNV-L1, WNV-L2, and USUV) from inactivated viral cultures were included in each nucleic acid extraction run. Before extraction, 500 *μ*l of MEM, 1x prepared with antibiotics (penicillin and streptomycin), L-Glutamine, and inactivated foetal bovine serum, was added to each tube and mosquito pools were crushed. A volume of 200 *μ*l of this mix was used for RNA extraction. For flavivirus detection, a triplex real-time reverse transcription-polymerase chain reaction (rRT-PCR), which simultaneously detects and differentiates WNV-L1, WNV-L2, and USUV, was employed [25]. For the characterization of USUV-positive pools, we used two overlapping generic RT-nested-PCR to detect flavivirus genome on the NS5 gene region [26, 27]. The amplified products were visualized by electrophoresis on a 1.5% agarose gel and were purified using ExoSAP-IT kit (GE Healthcare). The purified DNA was Sanger-sequenced in duplicates in both directions using the same primer sets of the RT-nested-PCR assays. Analysis of the sequences and assembly was performed by using SeqMan software (DNASTAR LASERGENE Software). The genome sequence obtained in this study was submitted to GenBank database (accession no. ON838179). Multiple alignments were performed using ClustalW program, and the best-fitting evolutionary model was based on those defined using JModeltest2 [28] on the basis of the Akaike information criterion. Tree reconstruction was carried out with Mega 11 [29]. Two phylogenetic analyses were performed on the basis of 1030 and 238-nucleotide fragment of the NS5 gene using the maximum likelihood (ML) method, Kimura 2-parameter model, and the general time reversible model [30]. Initial trees for the heuristic search were obtained automatically by applying the Neighbor-Join and BioNJ algorithms to a pairwise distances matrix estimated using the maximum composite likelihood approach and then selecting the topology with a higher log likelihood value. All positions containing gaps and missing data were eliminated. Bootstrap coefficients were calculated for 1000 replicates.

### 2.4. Detection of Flavivirus Antibodies in Wild Birds

Bird sera were screened to detect flavivirus antibodies with the blocking ELISA kit INGEZIM West Nile COMPAC (INgenasa, Spain), following the manufacturer's protocol. Although this ELISA kit is highly sensitive for WNV, cross-reacting antibodies to other closely related flaviviruses such as USUV may also react [18, 31]. Thus, in order to specifically detect USUV-neutralizing antibodies and differentiate from other cross-reacting flaviviruses, ELISA-positive and doubtful sera were subsequently analysed with a micro-virus-neutralization test (micro-VNT) in 96-well microtitre plates as described in [31]. Ten bird serum samples lacked enough volume so that they could not be analysed by micro-VNT.

Micro-VNTs were performed in parallel against USUV, WNV, and Bagaza virus (BAGV) as described in [31]. Viral strains used in this assay were USUV SAAR-1776 (accession no. AY453412), WNV E101 (accession no. AF260968), and BAGV Spain/RLP-Hcc1/2010 (accession no. KR108244). Samples yielding neutralization (complete absence of CPE) at 1 : 10 or higher was scored as positives. To confirm antibodies as specific for a determined virus, antibody titre should be at least fourfold higher for a given flavivirus over the rest [32]. If that threshold was not reached, the specific flavivirus that caused the infection could not be determined. Sera were considered USUV-positive only if the positive/doubtful result by ELISA was confirmed by VNT. In this study, we focused on the USUV results, as the WNV outcomes are part of a different survey (unpublished data).

### 2.5. Statistical Analysis

The estimated prevalence of USUV in mosquitoes and the corresponding 95% CI from variable pool size and perfect tests were calculated with the *EpiTools* epidemiological calculator method (https://epitools.ausvet.com.au/ppvariablepoolsize). This method estimates prevalence and confidence limits for variable pool sizes and assumes 100% test sensitivity and specificity [33].

The prevalence of flavivirus antibodies detected by ELISA and USUV-specific antibodies determined by micro-VNT were estimated as the ratio of positives from the total number of samples analysed, with the exact binomial confidence intervals (CI) of 95% based on the score method [34]. This prevalence should be considered as underestimated, or at least as the minimum detectable prevalence, taking into account that the potential cross-reactions detected with the ELISA test could not detect all sera with USUV antibodies. Descriptive statistical analyses were conducted in IBM SPSS Statistics for Windows, Version 26.0 (Armonk, NY: IBM Corp.).

## 3. Results and Discussion

### 3.1. Results

Overall, we collected 6,004 (5,862 female and 142 male) mosquitoes belonging to four genera and 13 species: *Cx. pipiens* (*n* = 4,622)*, Culex theileri* (*n* = 535)*, Culex perexiguus/univittatus* (*n* = 300)*, Aedes caspius* (*n* = 276)*, Aedes vexans* (*n* = 90)*, Anopheles atroparvus* (*n* = 90)*, Culiseta longiareolata* (*n* = 47)*, Culiseta annulata* (*n* = 14)*, Aedes pulcritarsis* (*n* = 13), *Aedes berlandi* (*n* = 10)*, Aedes echinus* (*n* = 4), and *Culiseta subochrea* (*n* = 3). The sequences obtained from three specimens of the *Univittatus* subgroup confirmed the presence in the same sampling site (B) (Figure 1) of *Cx. univittatus* (accession numbers: LC659916-7) and *Cx. perexiguus* (accession numbers: LC659918).

**Figure 2:**
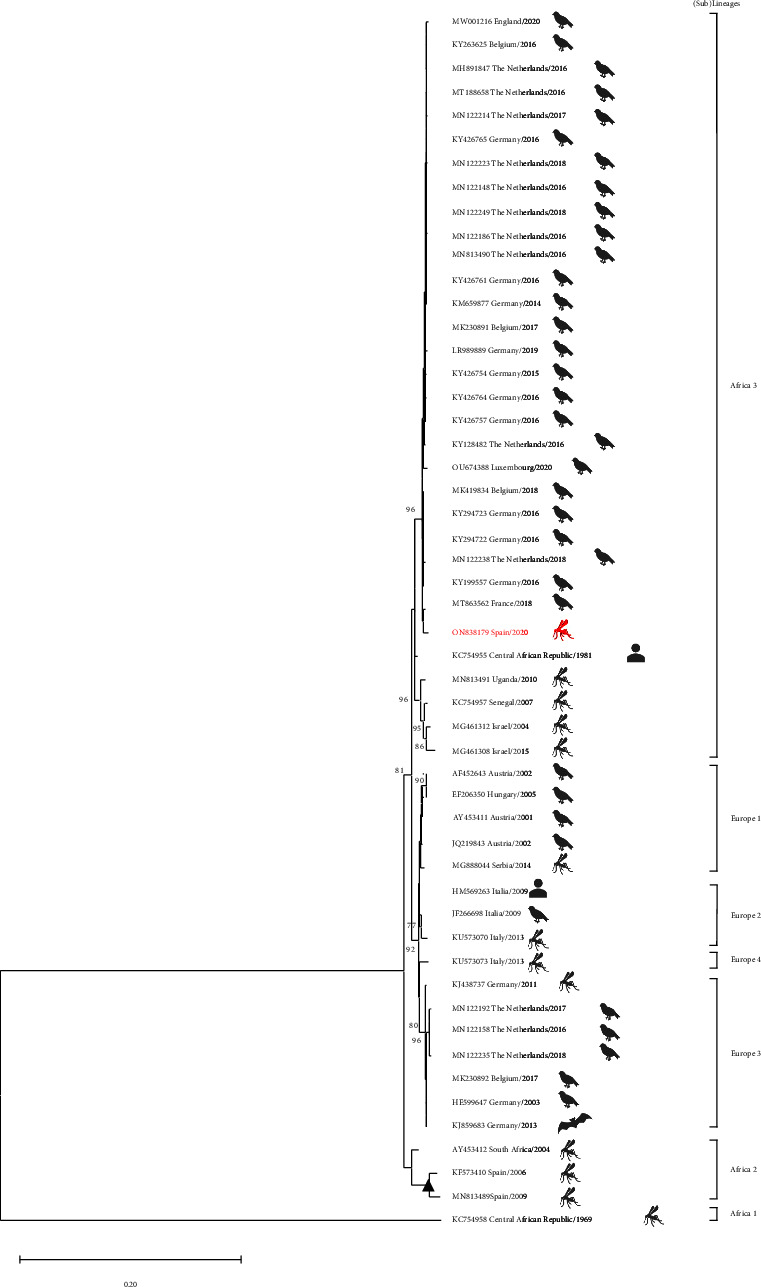
Phylogenetic analysis of a 1,030-nucleotide fragment of the NS5 gene using the maximum likelihood method and Kimura 2-parameter model. This analysis involved 52 nucleotide sequences and a total of 1,028 positions in the final dataset. Sequence obtained in this study is highlighted in red (accession no. ON838179), and the Spanish USUV sequence obtained from mosquitoes is marked with a black triangle. The percentage of replicate trees in which the associated taxa are clustered together in the bootstrap test (1000 replicates) is shown next to the branches. Only bootstrap >75% is shown. Taxon information indicated in the branches includes the country of origin, isolation/detection year, host, and GenBank accession number. USUV genetic sublineages are indicated on the right.

All the female mosquitoes were grouped in 438 pools. Of these, two pools of *Cx. pipiens* mosquitoes were positive for USUV by rRT-PCR, one captured on 16th August and one on 25th October in the same sampling site (B) (Figure 1). The vector infection rate for USUV in *Cx. pipiens* species was 4*e*-04% (95% CI: 1*e*^−04^–0.0014). A fragment of 1,030 nucleotides of the USUV NS5 gene was obtained from one *Cx. pipiens* pool (accession no. ON838179). The phylogenetic analysis showed the obtained sequence clusters with other sequences belonging to the USUV Africa 3 lineage, previously detected in Africa and several European countries (Figure 2). Indeed, the sequence obtained in *Cx. pipiens* mosquitoes from Extremadura differs from previous Spanish sequences obtained from mosquitoes captured in Catalonia and Andalusia (in 2006 and 2009 from *Cx. pipiens* and *Cx. perexiguus*, respectively). Besides, our sequence even differs in the analysis of the 238-nucleotides fragment of the overlapping sequence obtained from Spanish song thrushes in 2012 (accession no. KC437386) (S1).

Out of 1,413 wild bird sera, 103 were positive by competition ELISA (7.29%, 95% CI: 6.05–8.76) and 77 sera (5.45%, 95% CI: 4.38–6.76) were assigned as doubtful. USUV-specific antibodies were confirmed by micro-VNT in 17 birds (1.2%, 95% CI: 0.75–1.92), ten males and seven females from eight different species, three of them found infected for the first time in Europe (Table 1). Neutralizing USUV titres ranged from 1 : 10 to 1 : 320, detecting 7 samples ≤ 1 : 40.

Most of the positive birds were adults, except for four juvenile females captured in 2020: two Azure-winged magpies (*Cyanopica cyanus*), one great tit (*Parus major*), and one red avadavat (*Amandava amandava*) (Table 1). Seropositive birds were detected in all sampling areas (Figure 1).

### 3.2. Discussion

The detection of USUV in mosquitoes and wild birds from urban and rural areas has proven to be effective both to assess the epidemiological situation after outbreaks and to forecast possible future risks [35–37]. The survey carried out in this study with the analysis of mosquitoes and birds confirmed active USUV circulation in areas close to urban centers from southwestern Spain, providing new evidence that adds up to previous studies that had detected WNV and USUV antibodies in horses and birds in the same area in 2017–2019 [19, 20].

Mosquito pools positive for the presence of USUV belong to *Cx. pipiens* species, and they were captured in late summer-early autumn at the sampling site B (Figure 1). In other European countries, USUV-positive mosquitoes have usually been detected between June and October [12, 15].

Different mosquitoes, mainly ornithophilic species of the *Culex* genus, are known to participate in the transmission of USUV to wild or captive avifauna [1]. *Cx. pipiens* is a proven vector for USUV, and this virus has already been detected in specimens of this mosquito species in Europe [1], including Spain [14]. *Culex pipiens* was the most abundant mosquito species captured in this study, mainly in urbanized areas, the mosquito species with the greatest distribution in Extremadura [38]. It has been suggested to play a key role in the epizootic transmission of pathogens to humans [39]. So, it should be taken into account in control programs due to its potential for transmitting zoonotic flaviviruses in areas close to urban environments.

In Spain, USUV sequences of Africa 2 lineage have been detected in mosquitoes from Catalonia (*Cx. pipiens*, 2006) and Andalusia (*Cx. perexiguus*, 2009) [14, 15] and other unclassified USUV lineage in *T. philomelos* in 2012 [17]. Our study represents the first clear identification of USUV Africa 3 lineage in Spain. USUV strains of Africa 3 lineages were previously detected in Europe in *Cx. pipiens* mosquitoes in southern France [7, 12] and in common blackbirds (*Turdus merula*) from Germany (2014), Belgium (2016) [11], France (2018) [12], Austria (2017) [40], Czech Republic (2018) [35], the Netherlands [41, 42], the United Kingdom (2020) [43], and Luxembourg in 2020 [44].

In November 2012, USUV was detected in a song thrush (*Turdus philomelos*) from a die-off of ≈10 birds on a hunting estate in southern Spain [17]. Here, we found six seropositive common blackbirds. Considering that common blackbirds showed high morbidity and mortality rates due to the USUV infection in different European countries [45], possibly *Turdus* sp. may represent a key host in the USUV epidemiology. However, the high number of seropositive individuals from this species may raise other questions: has there been a trade-off between the adaptation of the virus to this species and its increased survival of the disease? Or is the Africa 3 lineage less pathogenic to blackbirds than other lineages circulating in Europe? Accordingly, the histological lesion severity in common blackbirds for the two identified lineages (Europe 3 and Africa 3) was compared, showing no significant differences [41]. Thus, it is likely that both lineages may produce a similar pathogenetic effect in naturally infected animals.

Spain is the southernmost country in Europe where USUV has been detected, and the sequence obtained is closely related to another sequence from France detected in 2018 [12]. As both sequences differ from other variants circulating in northern countries, this clade may represent an independent introduction event followed by geographical spread. As new sequences are continuously published, future analyses will allow us to better understand the spread dynamics of the USUV Africa 3 lineage in Europe and further decipher its evolutionary history.

Outside Europe, the Africa 3 lineage of USUV has been detected in mosquito species of the *Univittatus* subgroup (*Cx. perexiguus*, *Cx. univittatus,* and *Cx. neavei*) from Senegal [46], Uganda [47], and Israel [48], in *Cx. perfuscus* from Senegal and the Central African Republic [46], and in *Ae. albopictus* from Israel (unpublished, GenBank: MG461308.1). The presence of *Cx. perexiguus* and *Cx. univittatus*, important vectors of WNV and USUV, has already been reported in Extremadura [18]. Here, we confirmed the presence of both species in the same areas, which may imply a higher risk of flavivirus transmission considering that WNV lineage 1 was detected in *Cx. univittatus* in Portugal [49], and WNV lineage 1 and USUV Africa 2 lineage were detected in *Cx. pipiens* and *Cx. perexiguus* species in Spain, respectively [15, 50]. The difficult morphological differentiation of adult specimens of the *Univittatus* subgroup, together with their possible presence in the same habitats requires special care in their identification during entomological surveys, at least in the south of the Iberian Peninsula.

USUV antibodies were detected in birds from all sampling sites, including locality A, which is 41 km away from the city of Badajoz. In previous transmission seasons, USUV-specific antibodies were found in birds and horses in several areas from the Extremadura region [18–20]. The data obtained in this study confirm the occurrence of local transmission in 2020, with probable overwintering from past transmission seasons after its introduction in the area, indicating the establishment of an USUV enzootic cycle in southwestern Spain. In Europe, evidence of USUV infection has been found in at least 93 bird species from 35 families [1]. With this study, we enlarge the list of USUV hosts, including woodchat shrike (*Lanius senator*), common nightingale (*Luscinia megarhynchos*), and red avadavat (*Amandava amandava*). In the case of the woodchat shrike and common nightingale, both species breed in the Iberian Peninsula but are trans-Saharan migrants [51, 52]. The red avadavat is a bird indigenous to Asia which was introduced in the south of the Iberian Peninsula and Extremadura more than 40 years ago [53].

Overall, more than 75% of the positive birds of this study are resident species (and some juvenile specimens) with limited geographical mobility, which strongly indicates endemicity and establishment of the USUV in the region, considering that specific antibodies against this virus have been detected in different species since 2017 [18, 20]. Furthermore, the observed USUV seroprevalence in the area (1.2%) is similar to previous studies in wild birds in nearby sampling areas (0.96%) [20] and in birds from rehabilitation centres (1.4%) [18].

Nevertheless, we are aware of the methodological limitations of the present study and these findings should be interpreted with caution. The ELISA kit employed for screening was developed specifically for the detection of antibodies against WNV. Nevertheless, and in the absence of a commercial ELISA kit for specific USUV detection, its cross-reactivity with antibodies directed to other flaviviruses led to think it may be useful in USUV surveillance studies [31], but with unknown and possibly underestimated efficacy.

USUV and WNV share environmental and ecological drivers. Thus, cocirculation is often observed in nature [9, 19, 54, 55]. Indeed, both pathogens circulate within the same vectors in Spain (i.e., *Cx. pipiens* and *Cx. perexiguus*) [14, 15]. Consequently, USUV should be included, together with WNV, in surveillance programs and differential diagnoses in humans and animals. This recommendation is supported owing to the fact that 16 of 17 positive samples were obtained from birds captured at less than 5 km of the city centre and that 12 of these samples correspond to resident birds. Although some USUV acute neurological human cases have been reported (e.g., encephalitis or meningoencephalitis) [2], in most cases, USUV (and WNV) infections are asymptomatic or cause mild clinical symptoms [1]. Therefore, the actual incidence of USUV infection in humans in the region is essentially unknown, and thus, its epidemiological status and actual burden in public health is likely underestimated. Further epidemiological studies about USUV and other flaviviruses are warranted, especially considering the current scenario of increasing urbanisation, where human activities, such as animal husbandry and intensive agriculture, greatly influence the dynamics of vector-borne diseases through their impact on the distribution of hosts [56] and mosquito species [57–59].

## 4. Conclusions

In summary, this is the first identification USUV Africa 3 lineage in Spain. The detection of USUV in mosquitoes by RT-PCR and USUV-specific antibodies in wild birds (especially in juveniles) is indicative of an active circulation of the virus and the possible establishment of its enzootic cycle in southwestern Spain. The circulation of USUV close to urban areas represents a public health threat that demands its inclusion in the differential diagnoses in patients with compatible symptoms. Therefore, it is highly advisable to establish integrated bird and mosquito survey programs in the region, targeting urban and rural areas with the aim to collect relevant data on the epidemiological scenario represented by emerging flaviviruses, including USUV, and its evolution and spread, which may reveal new foci, seasonality, and ecological niches of USUV.

## Figures and Tables

**Table 1:** Results obtained for positive/doubtful samples by ELISA and with Usutu virus-specific antibodies by micro-VNT in wild birds from Extremadura, Spain.

Sampling sites	Species name	Common name	Age	Behaviour	Sex	Date	ELISA test	USUV VNT titre
A: Asesera	*Lanius senator^∗^*	Woodchat shrike	Adult	Migratory	Male	18/06/2020	Doubtful	1 : 10
B: Rincón de Caya	*Turdus merula*	Blackbird	Adult	Native	Female	30/06/2020	Doubtful	1 : 160

C: Bótoa	*Delichon urbicum*	House martin	Adult	Migratory	Male	12/06/2020	Doubtful	1 : 40
	*Delichon urbicum*	House martin	Adult	Migratory	Female	12/06/2020	Doubtful	1 : 20
	*Luscinia megarhynchos^∗^*	Common nightingale	Adult	Migratory	Male	15/06/2020	Positive	1 : 80
	*Sylvia atricapilla*	Eurasian blackcap	Adult	Native	Female	15/06/2020	Positive	1 : 20
	*Sylvia atricapilla*	Eurasian blackcap	Adult	Native	Male	15/06/2020	Positive	1 : 40
	*Turdus merula*	Blackbird	Adult	Native	Male	15/06/2020	Doubtful	1 : 160
	*Turdus merula*	Blackbird	Adult	Native	Male	15/06/2020	Positive	1 : 160

D: Gévora	*Cyanopica cooki*	Azure-winged magpies	Juvenile	Native	Female	10/08/2020	Positive	1 : 10
	*Cyanopica cooki*	Azure-winged magpies	Juvenile	Native	Male	10/08/2020	Positive	1 : 40
	*Parus major*	Great tit	Juvenile	Native	Female	24/07/2020	Doubtful	1 : 80
	*Sylvia atricapilla*	Eurasian blackcap	Adult	Native	Male	16/07/2020	Positive	1 : 160
	*Turdus merula*	Blackbird	Adult	Native	Female	24/03/2021	Positive	1 : 320
	*Turdus merula*	Blackbird	Adult	Native	Male	24/03/2021	Positive	1 : 160
	*Turdus merula*	Blackbird	Adult	Native	Male	24/03/2021	Positive	1 : 80
E: Sagrajas	*Amandava amandava^∗^*	Red avadavat	Juvenile	Exotic	Female	12/01/2020	Positive	1 : 160

^∗^Species that have been detected with specific USUV antibodies for the first time in Europe.

## Data Availability

The data used to support the findings of this study are available from the corresponding author upon reasonable request.

## Supplementary Materials

Supplementary Materials Figure S1: phylogenetic analysis of a 238-nucleotide fragment of the NS5 gene using the maximum likelihood method and Kimura 2-parameter model. This analysis involved 43 nucleotide sequences. The sequence obtained in this study is highlighted in red (accession no. ON838179), the Spanish USUV sequence obtained from birds is marked with a black square, and that obtained from mosquitoes is marked with a black triangle. The percentage of replicate trees in which the associated taxa are clustered together in the bootstrap test (1000 replicates) is shown next to the branches. Taxon information indicated in the branches includes the country of origin, isolation/detection year, host, and GenBank accession number. The USUV genetic sublineages are indicated on the right.
