# Supplementary figures and images for: Identification of Usutu Virus Africa 3 Lineage in a Survey of Mosquitoes and Birds from Urban Areas of Western Spain
Source: Transbound Emerg Dis. 2023 Feb 22;2023:6893677. doi: 10.1155/2023/6893677 (PMC12016895; doi:10.1155/2023/6893677)

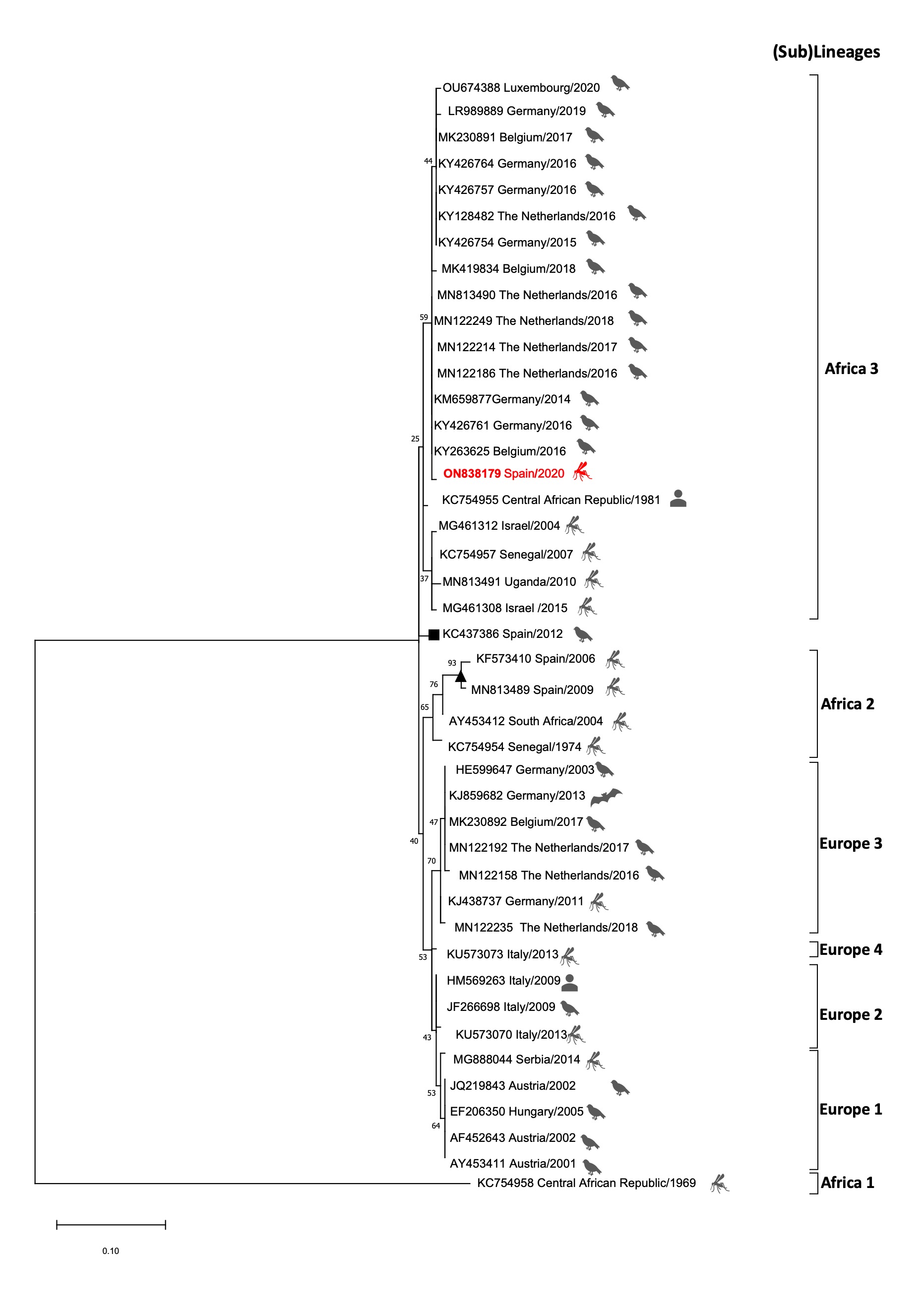

Supplement: Supplementary Materials — Figure S1: phylogenetic analysis of a 238-nucleotide fragment of the NS5 gene using the maximum likelihood method and Kimura 2-parameter model. This analysis involved 43 nucleotide sequences. The sequence obtained in this study is highlighted in red (accession no. ON838179), the Spanish USUV sequence obtained from birds is marked with a black square, and that obtained from mosquitoes is marked with a black triangle. The percentage of replicate trees in which the associated taxa are clustered together in the bootstrap test (1000 replicates) is shown next to the branches. Taxon information indicated in the branches includes the country of origin, isolation/detection year, host, and GenBank accession number. The USUV genetic sublineages are indicated on the right. [file 6893677.f1.jpeg]
